# ASSOCIATIONS BETWEEN LIFE COURSE ADVERSITIES AND SUBJECTIVE VIEWS ON AGING

**DOI:** 10.1093/geroni/igae098.4122

**Published:** 2024-12-31

**Authors:** Takeshi Nakagawa, Daisuke Ito, Saori Yasumoto

**Affiliations:** Osaka University, Suita, Osaka, Japan; Tokyo Metropolitan University, Tokyo, Japan; Osaka University, Suita, Osaka, Japan

## Abstract

Recent research indicates that how individuals perceive their own aging may be influenced by negative life events or adversities in early life, such as war (Korniek et al., 2024). However, little is known about whether and when the impacts of adversities become salient across the lifespan. This study examined the associations between life course adversities and subjective views on aging. Data was derived from a nationally representative survey of Japanese older adults aged 60 years and above. Life course adversities (including occurrence and age at first occurrence) were measured retrospectively in 2006. Subjective views on aging and covariates (including sociodemographic, social, and health-related factors) were measured in 1996. A total of 782 participants (60-84 years at baseline; female 54.2%) completed the two surveys. Of these participants, 88.9% experienced at least one adversity in their lifetime. We conducted multiple linear regression analysis to examine the associations of life course adversities, classified as childhood (ages 0-12), adolescence (ages 13-19), young adulthood (ages 20-34), midlife (ages 35-54), and older adulthood (ages 55+), with subjective views on aging. We found that, while individuals who experienced more adversities in older adulthood perceived their own aging as more negative (Beta = -.076, p = 0.035), those who experienced more adversities in childhood perceived their own aging as more positive (Beta =.095, p = 0.009). Results suggest that adversities at different ages may have differential effects on subjective experiences in late life. Further examinations are required to better understand the resilience and vulnerability of older adults.

